# Medical Domain of the Turkish Bath

**Published:** 1870-04

**Authors:** M. P. Hanson


Article II. — *Medical Domain of the Turkish Bath.* (Continued.) By M. P. Hanson, M.D.

In our former article we labored to show from physiological reasoning, and well ascertained facts, that the Turkish bath is the most natural, safe and powerful agent for the accomplishment of many indications of cure in the treatment of disease, viz.:

To equalize the circulation, and thus remove congestion.

Distribute nutrition.

Restore, and keep active all the secretions, and thus eliminate poison from the system.

If my conclusions are admitted, all physicians will be prepared to concede to the thermæ a wide range of usefulness in the prevention and cure of disease, and the experience of ages among the Eastern nations, and the more enlightened experience of the best medical authority of Ireland, England, France and Germany, for the last twelve years, amply justifies such a concession.

And, as a matter of fact, we find,

I. That the bath is of the greatest utility in all passive diseased states, wherever action is below par, as in the commencement of acute diseases; in the premonitory stages of fevers; and inflammations. In the congestive stage of eruptive diseases — measles, scarlet fever, small-pox, etc., whenever collapse takes place, and the symptoms show a retrocession of the fluids from the surface to the interior; whenever congestion of vital organs exists or is apprehended.

II. The Turkish bath will diminish, or prevent the liability to take infectious diseases. Heat is the greatest disinfectant known. Heat that will harden albumen (or cook an egg), will kill any animal poison, whether it be the natural secretions of animals, or the matter or effluvia of infectious diseases, as small-pox, syphilis, etc. Also, the liability to take infectious diseases often depends on a habitually sluggish condition of the kidneys, with scanty secretions. Now, the powerful revulsion to the surface, and drain of fluids and effete matter secured by the bath, eliminates the poison from the system.

III. In *Bright's disease* the condition of the patient begins to improve on his first introduction to the bath. Even in cases where the secretion of urine is almost suspended; where the system is saturated with urea, and dropsical effusion is general, and the brain begins to show signs of the irritation of the poison in the blood, the bath will correct the most troublesome symptoms more surely and quicker than any other means known. Even in cases where the kidneys are so far disorganized as to render a cure hopeless, the bath will cause the skin to take on a vicanous function, and it will, as Dr. Shrudichum declares, "throw out large quantities of uric acid, and rid the system of the effete and poisonous matter that should have been discharged by the kidneys," and thus prolong and make comfortable a life that, under any other treatment, would be very short and wretched in the extreme.

IV. *In diabetes* the functions of the skin are suspended, and extra duty thrown on the kidneys. The bath will restore the functions of the skin as nothing else will, the quantity of urine and its specific gravity begins to diminish immediately, the inordinate thirst disappears, sleep returns, the spirits revive, the pain in the loins and limbs subside, and the whole condition and appearance of the patient changes in many cases as by magic.

V. In nervous diseases of the heart, and in those connected with organic disease, angina, pectoris, hydro-pericardium, the hot room does actually quiet the circulation and improve the condition of the patient almost immediately on his entrance.

VI. In *bronchitis*, or irritative congestion of the windpipe and larynx, the bath cannot fail to be permanently useful, for this disease is usually only symptomatie of a morbid condition of the skin and digestive organs. In acute affections of the throat, even in croup and diphtheria, the bath will almost invariably save life.

VII. *In consumption* the bath will, on the clearest abstract grounds, as well as on the showing of facts, produce the greatest ratio of arrests of that terrible disease.

The tubercle producing products of imperfect digestion are drained out of the system ; internal congestions are removed ; the air-cells dilate ; the relaxed skin braced ; the appetite and power of appropriation increased ; night sweats checked, and sleep secured.

VIII. *In dropsies*, whether of the shut cavities or cellular tissue, the bath is the most direct and natural cure, for it appeals directly to the absorbents to furnish water to protect the surface, and the effused fluid is actually taken up and restored to the circulation and evaporated from the surface. I have seen many pounds of fluid taken out of the system in a single bath, and the actual measurement of the patient reduced, around the abdomen, three inches.

IX. In *chronic liver disease;* in enlargement, or simple congestion ; in the passage of gall stones ; and all liver obstructions, the bath will afford relief quicker, and at less expense to the system, than any other treatment.

X. In *gout* and *rheumatism* the bath is the remedy *par excellence.*

XI. In *diarrhœa* and *dysentery* the bath is the cure, as determining the fluids from the internal linings to the skin, thus relieving internal congestion, on which the disease depends.

XII. The bath will take down, summarily and safely, excessive obesity, while it will as surely promote the nutrition of the ill nourished, increasing the appetite in proportion as it increases the power of appropriations.

XIII. The bath is the truest and best anti-spasmodic in bilious colic ; in spasms of the bronchial tubes ; asthma, even in lockjaw, and tetanus. Between the combined effect of the hot room and the cold douche, spasms and cramps, of all sorts, will find a power to control them, by equalizing nervous action, and causing division of nervous excitability.

XIV. The Turkish bath will remove that very offensive personal odor, whether of the breath or the person, that attaches to some apparently cleanly and healthy people, and always, more or less, to the insane, giving that peculiar stench to asylums where they are congregated. This condition depends upon the accumulation of effete matter in the follicles and sebaceous glands of the skin. The bath, by flushing these drains, will remove the cause of insanity, in many cases, and make some people that are not insane fit for human society that never were before.

XV. *In uterine diseases*, whether suppressions or dysmenorrhœa, or that general failing of all the functions, known under chlorosis, the bath will invariably relieve, by removing the conditions on which the symptoms depend, whether of congestion of the organ, which is most frequently the case, or lack of development of the function. In either case, the bath is the power by which the function can be established and controlled.

But I have gone far enough into detail. No experienced physician, if he admits any conclusions, will fail to see that the medical domain of the thermæ is vast and important.

Dr. Carl Luther, of Berlin, says: "Formerly, when I received a patient, my first inquiry was to find the name of the disease. Since I have adopted the Turkish bath, I feel so sure of a cure I do not care to know the name of the disease."

Dr. Gosse, of Geneva, says: "The Turkish bath is the real panacea for the larger portion of the diseases that assail mankind."

Dr. I. L. W. Shudichum, of London, says: "In the Turkish bath I feel that I have put into my hands the most powerful and certain, and, at the same time, the most safe and agreeable therapeutic agent in existence."

Dr. Erasmus Wilson, of London, in an address to the British Medical Association, said: "We have thus presented to us the effects of the bath, applied to the skin:

"1st. An improvement of organic structure.

"2nd. An improvement of secretive function.

"3rd. An improvement in circulation and respiratory power.

"4th. An improvement in inervation and sensation."

"The bath that cleanses the inward as well as the outward man; that is applicable to every age; that is adapted to make health healthier, and alleviate disease, whatever its stage or severity, deserves to be adopted as a national institution, and merits the advocacy of all medical men; of those whose especial duty it is to teach how health may be preserved, and how disease may be averted."

Dr. John Balbirnie, of London, says: "If I were asked to give a brief and distinctive definition of the Turkish bath, I would say, it is that which claims the exclusive, pre-eminent power of physiologically opening the safety valves of the living mechanism; or developing a high activity of the depurating functions of the animal body, and so fulfilling the first grand indication for the cure of all diseases."

